# Commentary: Detailed Visual Cortical Responses Generated by Retinal Sheet Transplants in Rats With Severe Retinal Degeneration

**DOI:** 10.3389/fnins.2019.00471

**Published:** 2019-05-10

**Authors:** Michael Beyeler

**Affiliations:** ^1^Vision and Cognition Laboratory, Department of Psychology, University of Washington, Seattle, WA, United States; ^2^Institute for Neuroengineering, University of Washington, Seattle, WA, United States; ^3^eScience Institute, University of Washington, Seattle, WA, United States

**Keywords:** retinitis pigmentosa (RP), retinal sheet transplant, sight recovery, retinal degeneration, age-related macular degeneration (ARMD)

## 1. Background

Retinal degenerative diseases such as retinitis pigmentosa (RP) and age-related macular degeneration (ARMD) are among the leading causes of blindness in the world. These diseases are characterized by a progressive loss of photoreceptors and/or retinal pigment epithelium (RPE), leading to severe remodeling of the retinal circuitry (Marc et al., 2003) and a gradual loss of vision. However, cells in the inner retina that connect to the brain may remain functional throughout the disease (Santos et al., 1997). Therefore, if the diseased cells could be bypassed or replaced with new cells that connect to the functional part of the retina, it might be possible to restore vision in affected individuals.

Several treatment options are currently in development, such as micronutrient supplementation (Lavail, 2005) and gene therapy (Liu et al., 2011), including more controversial interventions, such as hyperbaric oxygen therapy (Vingolo et al., 1998, 2008). However, these interventions have to happen in early stages of the disease, before photoreceptors are irreversibly degenerated. In severe cases of RP and ARMD, the only FDA-approved treatment option are retinal prostheses (also known as “bionic eye” or “artificial retina”; see Weiland et al., 2016 for a recent review), which aim to evoke neuronal responses in surviving cells through electrical microstimulation—but their success has been limited to date.

Another approach is to replace diseased cells with healthy cells through transplantation. A reliable option for transplantation in several animal models is human fetal tissue, which is best transplanted in sheet form that contains the RPE (Seiler and Aramant, 2012). Although retinal sheet transplants (RSTs) can restore responses to flashes of light in the superior colliculus, neuronal responses at the level of visual cortex remain poorly understood.

## 2. Retinal Sheet Transplants Restore Vision in Rats

To address this issue, Foik et al. (2018) investigated the capability of RSTs to restore vision in pigmented transgenic line-3 rats. Following successful transplantation in the subretinal space between host degenerated retina and RPE (their Figure 1), the authors measured visually evoked responses in primary visual cortex (V1). Cells were first tested for visual responsiveness using flashes of light; then receptive fields were located using drifting gratings. Neuronal tuning curves were then compared to those of control degenerated animals that did not receive transplants, as well as to non-degenerated NIH and Long-Evans rats.

Foik et al. (2018) found that the number of visually responsive cells in V1 improved from 9% in control degenerated rats to 56% in transplanted rats, as compared to 87% in non-degenerated rats (their Figure 4). Moreover, visual sensitivity in transplanted rats had improved to the point where orientation, size, and spatial frequency tuning (but not temporal frequency tuning and contrast sensitivity) were statistically indifferent from V1 cells in sighted animals (their Figure 5). Feedforward input from the lateral geniculate nucleus, connectivity within V1, and feedback from higher visual areas were shown to be present in degenerated rats, but overall to a lesser extent than in transplanted and normal rats. Interestingly, long-range connectivity within V1 (beyond 300 μm) was reduced the most, whereas local connections remained largely unaffected. Furthermore, transplantation was able to restore the circuitry back to a level comparable to normal rats.

These findings suggest the presence of an activity-dependent plasticity mechanism that may lead to a reduction of cortical connections in the absence of visual input, but can be recruited to restore connectivity even months after vision loss. However, without further analysis it is unclear whether this plasticity could lead to functional cortical reorganization, or whether it mainly resembles the sort of corruptive retinal remodeling that occurs in later stages of photoreceptor disease (for a recent discussion on the subject, see Beyeler et al., 2017). Improved cortical responses could have also been due to neuroprotection of the remaining host photoreceptors, as the reactive change of Müller glial cells is a well-known obstacle for transplant integration (Hippert et al., 2016).

## 3. Retinal Sheet Transplants in Humans

An important open question is whether these findings will apply to transplantation in humans. Several clinical trials with RSTs have been underway since the late 1990s, but results have been mixed. Early clinical trials reported no adverse effects, but also no lasting vision improvement (Humayun et al., 2000). The best results were achieved in a Phase II clinical trial conducted in a group of ten patients (six RP and four ARMD) where the transplanted sheet included the RPE (Radtke et al., 2008): seven patients showed visual improvements after 1 year, with vision remaining the same in one RP patient, and vision decreasing in two others. In one subject, vision improved from 20/800 pre-operative to 20/160 at the 1-year mark, and remained stable at 20/200 over 5 years. Future research will have to show if these results can be repeated in a larger study sample.

The use of a transplant that includes the RPE is an attractive strategy, as healthy RPE cells should theoretically be able to restore all functions of the degenerated host RPE. However, the success of this strategy strongly depends on proper cell delivery and maintenance. Dissection of donor tissue has to be done very carefully, with minimal touching of the retina (Seiler and Aramant, 2012). Another drawback is the limited availability of fetal donor tissue. In the future, it may be possible to differentiate retinal and photoreceptor progenitors directly from embryonic stem cells—without the need for fetal tissue. To this end, a Phase I trial involving a stem cell-derived RPE patch just recently showed significant vision improvement in two ARMD patients (da Cruz et al., 2018).

In conclusion, this study represents an essential step toward determining the suitability of RSTs as a treatment option for severe retinal degeneration. However, much work remains to be done until this is a viable treatment option in humans. Although availability of fetal tissue will likely remain limited, much could be learned about the cellular mechanisms of sight restoration through this line of research, thus giving hope that the blinding effects of incurable eye disease could one day be reversed.

## Author Contributions

The author confirms being the sole contributor of this work and has approved it for publication.

### Conflict of Interest Statement

The author declares that the research was conducted in the absence of any commercial or financial relationships that could be construed as a potential conflict of interest.
